# Mucoadhesive Nanocarriers as a Promising Strategy to Enhance Intracellular Delivery against Oral Cavity Carcinoma

**DOI:** 10.3390/pharmaceutics14040795

**Published:** 2022-04-05

**Authors:** Manisha Pandey, Hira Choudhury, Jenifer Ngu Shao Ying, Jessica Foo Sze Ling, Jong Ting, Jocelyn Su Szhiou Ting, Ivory Kuek Zhia Hwen, Ho Wan Suen, Hazimah Syazwani Samsul Kamar, Bapi Gorain, Neha Jain, Mohd Cairul Iqbal Mohd Amin

**Affiliations:** 1 Department of Pharmaceutical Technology, School of Pharmacy, International Medical University, Kuala Lumpur 57000, Malaysia; 2 School of Pharmacy, International Medical University, Kuala Lumpur 57000, Malaysia; ngushaoying@gmail.com (J.N.S.Y.); jessicafoo71@gmail.com (J.F.S.L.); jong.ting_98@hotmail.com (J.T.); jocelynsu28@gmail.com (J.S.S.T.); ivorykzh@gmail.com (I.K.Z.H.); howansuen1999@gmail.com (H.W.S.); hazimahsyazwani15@gmail.com (H.S.S.K.); 3 Department of Pharmaceutical Sciences and Technology, Birla Institute of Technology, Mesra, Ranchi 835215, India; bapi.gn@gmail.com; 4 Department of Pharmaceutics, Amity Institute of Pharmacy, Amity University, Noida 201303, India; ngulati88@gmail.com; 5 Centre for Drug Delivery Technology, Faculty of Pharmacy, Universiti Kebangsaan Malaysia, Jalan Raja Muda Abdul Aziz, Kuala Lumpur 50300, Malaysia; mciamin@ukm.edu.my

**Keywords:** oral cancer, nanocarriers, mucoadhesion, targeted drug delivery approach, cytoplasmic delivery, improved efficacy

## Abstract

Oral cancer, particularly squamous cell carcinoma (SCC), has posed a grave challenge to global health due to its high incidence, metastasis, and mortality rates. Despite numerous studies and favorable improvements in the therapeutic strategies over the past few decades, the prognosis of this disease remains dismal. Moreover, several drawbacks are associated with the conventional treatment; including permanent disfigurement and physical impairment that are attributed to surgical intervention, and systemic toxicity that results from aggressive radio- or chemotherapies, which impacts patients’ prognosis and post-treatment quality of life. The highly vascularized, non-keratinized oral mucosa appears as a potential route for cytotoxic drug administration in treating oral cancer. It acts as a non-invasive portal for drug entry targeting the local oral lesions of the early stages of cancer and the systemic metastasis sites of advanced cancer. The absorption of the poorly aqueous-soluble anti-cancer drugs can be enhanced due to the increased permeability of the ulcerous mucosa lining in the disease state and by bypassing the hepatic first-pass metabolism. However, some challenges in oral transmucosal drug delivery include the drugs’ taste, the limited surface area of the membrane lining the oral cavity, and flushing and enzymatic degradation by saliva. Therefore, mucoadhesive nanocarriers have emerged as promising platforms for controlled, targeted drug delivery in the oral cavity. The surface functionalization of nanocarriers with various moieties allows for drug targeting, bioavailability enhancement, and biodistribution at the site of action, while the mucoadhesive feature prolongs the drug’s residence time for preferential accumulation to optimize the therapeutic effect and reduce systemic toxicity. This review has been focused to highlight the potential of various nanocarriers (e.g., nanoparticles, nanoemulsions, nanocapsules, and liposomes) in conferring targeting, solubility and bioavailability enhancement of actives and mucoadhesive properties as novel tumor-targeted drug delivery approaches in oral cancer treatment.

## 1. Introduction

Oral cancer is developed in the floor of the mouth, buccal mucosa, sublingual area, retromolar trigons, anterior two-thirds of the tongue, hard palate, upper and lower alveolar ridges, and the lips [1]. According to the data that are provided by the World Health Organization (WHO), it is estimated that there are approximately 657,000 new oral cancer cases each year [2]. Roughly 90% of oral cancer is associated with the differentiation of the squamous cells arising from the mucosal epithelium, a group of conditions which are known as the oral squamous cell carcinomas (OSCCs) [3]. OSCC represents 2 to 3% of all human cancer cases and it is often diagnosed in the advanced stage, stage III, or stage IV [4]. A total of 177,384 patients with oral squamous cell carcinomas died in 2018 [4]. Overall, it has been observed that only 50% to 60% of all of the individuals who suffer from OSCC have a chance of five years’ survival [5].

Human papillomavirus (HPV), tobacco abuse, and heavy alcohol use are the critical risk factors for OSCC [6,7,8,9,10,11]. The treatment of OSCC varies depending on the cancer’s stage [12]. In the early stage of OSCC, surgery or radiotherapy can be used alone as a treatment option [12], while in advanced stages, surgery and/or radiotherapy can be used with the addition of chemotherapy as an adjuvant [12]. Chemotherapy is being added to the treatment regimen as it can improve overall survival [12]. However, chemotherapy can result in multi-drug resistance (MDR) and diverse side effects, such as myelosuppression, nausea and vomiting, cutaneous reaction, oral mucositis, extravasation, and anemia, all of which cause a reduction in the patient’s quality of life [13]. Chemotherapeutic agents, such as cisplatin (CDDP) and fluorouracil (5-FU), are commonly reported alongside adverse effects. CDDP is often reported alongside gastrointestinal toxicity, nephrotoxicity, and neurotoxicity [12]. Besides this, MDR causes healthy cells to have high levels of drug expulsion, which in turn results in toxic manifestations that are associated with chemotherapy [14,15]. Surgery resection is the mainstay treatment of OSCC; however, it can lead to permanent disfigurement. Furthermore, patients may suffer from pulmonary infection and wound infection complications without proper perioperative care [16].

For managing OSCC, intensity-modulated radiotherapy is predominantly used in order to administer sharp and intense radiation to the target without damaging the surrounding tissues. However, side effects such as hair loss, trouble swallowing, and tooth decay are the most frequently encountered complications that lead to functional limitation and further impaired the quality of life for the patient [17]. Considering the limitations of the currently available treatment options, localized drug delivery has emerged to be an excellent alternative as it provides efficacious treatment by the cytoplasmic delivery of a drug for a specific region in the oral cavity without undesirable treatment-induced complications. Nanomedicine is a novel field that has been formed by the intersection of nanotechnology with physics, biology, mathematics, and medicine [12]. To overcome the debilitating difficulties that are caused by the currently available anticancer treatment, the novel nanocarrier tools from the nanotechnology-based system have been introduced for a site-specific drug accumulation of the therapeutic agents for cytoplasmic delivery and desired pharmacological action [15]. The nanocarriers have a nanometric size range, from 10 to 500 nm, enhancing the drug dissolution at the site of action due to an enhanced surface-to-volume ratio [18]. The anatomy of the oral mucosa displays rich vascularity and blood supply, which can be considered as a potential site for drug delivery in treating OSCC. However, the secreted saliva in the oral cavity forms a thin film coating throughout the buccal mucosa lining, limiting the drugs’ penetration and flushing out the drugs. Therefore, devices possessing mucoadhesion are necessary for better-localized adhesion and cytoplasmic drug delivery in the oral cavity. Mucoadhesion at the site of the oral cavity prolongs the time of contact between the oral mucosal membrane and the drug formulations [19]; mucoadhesion is defined as the adhesion between one mucosal surface with another mucosal or non-mucosal surface by the interfacial force for an extended period [20]. Mucoadhesive nanocarriers work by entrapping chemotherapeutic drugs into their matrix and they can be used passively or with iontophoretic treatment [21]. The mucoadhesive nanoparticle prolongs the contact time between the drug formulation and the oral mucosa, enhances the chemotherapeutic drug’s bioavailability, the onset of action, the drug’s penetration in the cytoplasm, and, ultimately, the therapeutic efficacy. Therefore, the present review has been focused on the usage of mucoadhesive nanocarrier in treating OSCC and its benefits compared to other conventional oral cancer treatment options. In addition, recent approaches to oral cancer diagnosis and treatment with nanocarriers and the benefits of mucoadhesive nanocarriers are also discussed in this review.

## 2. Overview of Oral Mucosa

### 2.1. Anatomy, Physiology, and Permeability of Oral Mucosa

The human oral mucosa has a total surface area of approximately 200 cm^2^, covering most of the oral cavity area that is demarcated by the lips, cheeks, soft and hard palates, and floor of the mouth. It consists of two layers: a superficial, avascular, stratified squamous epithelium of variable thickness and an underlying vascular layer of a connective tissue component, known as lamina propria, that is separated by a basement membrane. The oral stratified squamous epithelium can be further divided into the keratinized epithelium (the gingival mucosa and hard palate), which is relatively impermeable to water and demonstrates a barrier function, and the non-keratinized epithelium (the soft palate, buccal and sublingual mucosae) that is more permeable to water [22,23,24]. The keratinized epithelium is constituted by four distinct layers: the basal, prickle, granular, and keratinized layers, which resemble the different levels of cell differentiation. Rapidly proliferating basal keratinocytes are found at the basal layer, followed by partially differentiated keratinocytes which are found superficially, and terminally differentiated keratinocytes from the keratinized layer [22,25].

The permeability characteristic of the surface of the oral mucosa determines the penetration and absorption of the drug. Thus, it is essential that appropriate drug preparations are formulated in order to easily cross the underlying membranes for the desired pharmacological action [23]. The permeability of the different oral mucosa regions is based on the degree of keratinization and relative thickness of the mucosal membranes [25]. The keratinized mucosa possesses lower permeability that assists it to act as a site for the topical effect of highly potent drugs. In contrast, the non-keratinized mucosa is relatively more permeable, increasing the absorption of drug molecules into the plasma and contributing to systemic therapeutic effects [22]. Generally, the permeability is the lowest in the gingiva and hard palate, followed by the buccal mucosa, while the sublingual mucosa has the highest permeability [22,24].

As for drug diffusion across oral mucosa, this process involves three mechanisms: passive transcellular and paracellular diffusion, carrier-facilitated transport, and endocytosis/exocytosis [22]. Besides this, the drug’s characteristics also affect the extent of its permeability, with the drugs which confer high lipophilicity and have a low molecular weight (less than 75 to 100 Da) exhibiting the optimum penetration properties [22,24].

### 2.2. Permeability Barrier to Oral Mucosal Drug Absorption

The permeability barrier exists due to the formation of membrane-coated granules (MCGs) at the apical cell surfaces during suprabasal cell differentiation. The permeability barrier releases lipophilic substances into the intercellular spaces at the upper layers of the epithelium [22,24]. The lipophilic materials dampen the passage of hydrophilic substances across the epithelium, while the high hydration level within the connective tissue provides some resistance to lipophilic materials. Thus, the epithelium acts as the main barrier to permeability [22]. As discussed, the keratinized and non-keratinized epithelia have different levels of permeability due to the presence of different MCG lipids [24]. The MCG lipids of the keratinized epithelium include sphingomyelin glucosylceramides, ceramide, and other non-polar lipids; thus, it is relatively less permeable. In comparison, the main MCG lipid components for the non-keratinized epithelium consist of cholesterol, cholesterol esters, and glycosphingolipids, demonstrating its higher permeability [24].

Other factors that may affect the permeability of the mucosa include the amount of salivary flow, in which an increase in salivary secretion may decrease the mucosa’s permeability [24]. The action of enzymes such as dehydrogenases, carboxypeptidases, and aminopeptidases in the oral fluid can influence the absorption and therapeutic effect of the drugs [25]. The mucus layer is a physical barrier to drug permeation, whereas the mucins act as a lubricant and form a gel-like structure within the oral epithelium, affecting the drug delivery by the buccal route [24,25]. The basement membrane can also hinder the permeation of specific therapeutic agents such as chlorhexidine and beta-blockers [24].

### 2.3. Oral Cavity as a Site for Drug Delivery in Oral Cancer

Instead of presenting a barrier to penetrating therapeutics, oral mucosa is considered as a potential route for drug administration that is intended for different disease conditions, particularly the precancerous stage of oral cancer [22]. Potentially malignant oral cancer lesions are displayed as morphologically distorted tissue in clinical examination, for which the chance of malignant transformation is higher than its normal counterpart. Histologically, potentially malignant oral lesions show dysplastic features and could be premalignant. Approximately 20% of all oral dysplastic lesions can undergo a malignant transformation, of which 90% are squamous cell carcinomas [22,26]. Targeted oral delivery enables the accumulation of the drug in the premalignant oral lesion and increases the cytotoxic effect of the therapeutic agents [22]. Besides this, the alteration of the mucosal barrier and the defective vasculature endothelial junction in the cancerous state of oral mucosa also enhances the permeability of drugs to the malignant cells [22]. Furthermore, the systemic absorption of the cytotoxic drug through the non-keratinized layer of the oral mucosa makes it possible to treat the advanced tumor stage. To optimize treatment and minimize the side effects of cytotoxic drugs, distinct penetration and satisfactory drug retention profiles are required in order for oral mucosal delivery to achieve a desirable therapeutic effect for oral dysplastic lesions [22]. Thus, a connecting section of this present article has been included in order to emphasize the importance of nano carrier-based deliveries in the improvement of the therapeutic/diagnostic outcomes of therapeutics.

## 3. Conventional Treatments and Their Limitations

Current treatment regimens for oral cavity cancer vary according to the cancer’s stage, patient’s co-morbidity, and treatment’s acceptability [27,28]. However, there are many drawbacks stemming from the current treatments. Patients may suffer disfigurement, decreased swallowing ability, and speech trouble after surgery and radiotherapy [29]. Conventional systemic chemotherapy includes tablets and injections and lacks cancer cell selectivity [30], resulting in severe and undesirable effects, such as nephrotoxicity, neurotoxicity, gastrointestinal toxicity, and hair loss [30,31]. Chemotherapy is also susceptible to cancer cell efflux pumps, leading to multi-drug resistance [30,32]. The characteristics of cytotoxic drugs, such as poor aqueous solubility, low apparent permeability, and poor bioavailability, have hindered treatment efficacy [30]. Therefore, localized drug delivery options targeting the oral lesion serve as a potential treatment alternative. However, the conventional localized treatment for diseased oral mucosa faces obstacles like limited retention time and drug exposure, unpalatable taste, and enzymatic degradation [22]. Thus, novel formulation approaches with solutions for longer retention at the site of the cancer can help to overcome the associated limitations of the conventional deliveries.

## 4. Nanocarriers in Oral Cancer Detection and Treatment

**Figure 1:**
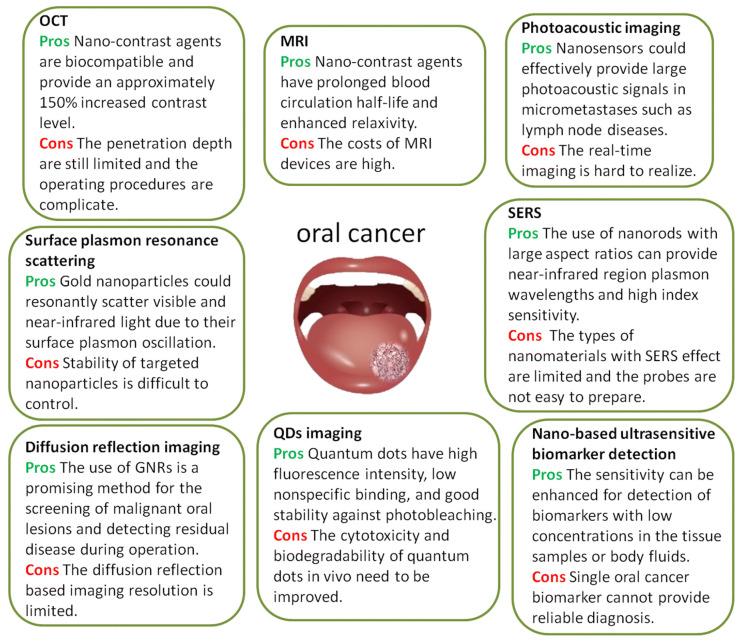
The pros and cons of different nanotechnology for bioimaging and biomarking [33].

The early detection of cancer is essential for its effective treatment and the survival of the patients, and this includes OSCC. Although a scalpel biopsy is the gold standard for oral cancer diagnostic procedures, it is often invasive, which may cause anxiety and discomfort in patients [33]. In addition, OSCC has a high chance of recurrence since this scalpel biopsy cannot detect small numbers of genetically abnormal cells in the body [33]. Therefore, improvisation has been carried out by encouraging non-invasive tools such as toluidine blue (TB) staining, auto-fluorescence (VELscope), and chemiluminescence (ViziLite) in the diagnosis of OSCC [31], where saliva acts as the easily accessible sample for oral cancer detection as it is more convenient and cost-effective [31]. Nanotechnology-based delivery tools contain particles within the nanometer size range [22,31], which are widely used in various cancer detection and diagnosis procedures and also in disease monitoring. There are two types of nanotechnology-based detection and diagnosis methods that have been introduced, viz: nano-based molecular imaging and nano-based biomarker imaging [33]. For instance, to detect cancer cells, MRI contrast agents are injected into patients’ bodies. Usually, MRI contrast agents such as gadolinium (Gd) complexes with diethyl triamine-penta-acetic acid (Gd-DTPA) or tetra azacyclododecane-1,4,7,10-tetra-acetic acid (Gd-DOTA) are widely used for this purpose [33,34]. Although they have high distribution, they are not tumor-specific. The chances of their accumulation in tumors are also less as they have a short half-life. Thus, nanocarriers are introduced in MRI contrast agents. These nano-contrast agents increase the specificity, prolong the half-life, and enhance the permeability and retention effect on the tumor tissues [22,31]. An example of this can be found in a study that was carried out by Antian et al., which showed that Omn-nanoparticles (Omn-NP) could be a promising MRI contrast agent for detecting and diagnosing oral cancer cells [33]. Since Omn-NP is tumor-specific and involves an effective delivery system, it accumulates in the affected areas, providing a longer half-life and thus higher chances of detection [31]. Similarly, optical coherence tomography (OCT) is a radiographic imaging modality that measures tissue stiffness in order to diagnose and detect oral cancer cells. This is a non-invasive and non-destructive test with high penetration up to 2 mm in depth in the epithelial layer and basement membranes [31]. This modality is commonly used for early oral cancer detection and oral dysplasia monitoring. However, the capability of OCT is limited due to the low contrast between neoplastic and normal tissues [33]. This limitation can be overcome by introducing gold (Au) NPs and biocompatible contrast agents [33,35]. For instance, PEG conjugates that are Au-NP clustered with acid-cleavable linkers can be used to detect mildly acidic tumor environments. The clustered Au-NP will be hydrolyzed and dispersed when mildly acidic oral tumors are detected, generating a reduced scattering intensity and fast Brownian motion, further providing a high-resolution OCT image [35]. The application of nanotechnology has shown several pros and cons, Figure 1 illustrates the pros and cons of different nanotechnologies that are used for bioimaging and biomarking.

Alternatively, different nanocarriers including liposomes, micelles, polymeric NPs, dendrimers, hydrogels, and others have portrayed tremendous opportunities in cancer therapies, including those for oral cavity carcinomas, due to their beneficial effects that have been proven in various research [36]. There are 51 types of nanomedicines that were approved by the Food and Drug Administration (FDA) in 2016, a figure which signifies the promising results of NPs in drug delivery systems [37]. For both local and systemic drug deliveries, the sublingual and buccal mucosal regions are more commonly chosen as these regions are highly vascularized, specifically for oral cancers [38]. Various dosage forms such as sprays, tablets, films, and patches incorporate NPs for therapeutic benefits [38]. Among all of the types of nanocarriers, the hydrogel is an excellent carrier that is broadly used in pharmaceutical applications and is widely studied for its positive effects on mucosal drug delivery [39].

Drug resistance is one of the main issues in the drug delivery of chemotherapeutic agents; however, this can be overcome by using this nanotechnology-based drug delivery system through encapsulation, attachment, and the conjugation of drugs or therapeutic biological products to nanocarriers [40,41].

## 5. Mechanism of Cytosolic Delivery of Drugs

The cellular internalization of nanocarriers is governed by their surface characteristics, size, and shape. The internalization can occur through different pathways; however, endocytosis signifies an attractive strategy for cellular internalization. The entrapment of a nanocarrier after its internalization in the endosomal compartment also represents an additional barrier for targeted drug delivery to the specific cell site. Moreover, translocation to the cytoplasm is prevented by the membrane of the lysosome and endosome. The therapeutic drug may undergo enzymatic degradation in the lysosomes. Therefore, different approaches are explored to enhance cytosolic delivery. In one approach, specific microenvironments such as pH, redox, and enzymes in the compartment have been found to facilitate drug release. However, the second approach emphasizes disrupting the endosomal membrane either via membrane-disrupting peptides or endosmotic polymers [42].

**Figure 2:**
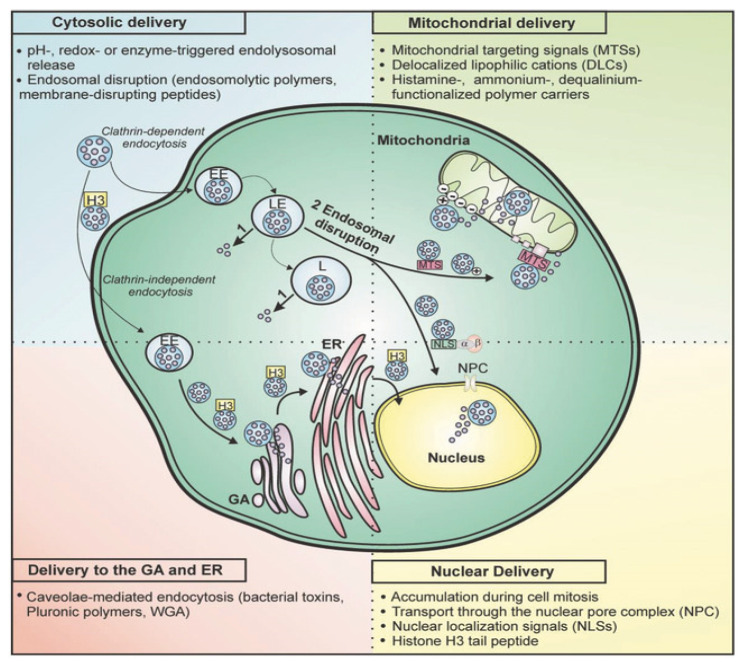
Overview of approaches that have been developed to control intracellular delivery of nanocarriers. Reprinted from ref. [43] with permission from John Wiley & Sons (2017).

Nanocarriers protect the drug molecules from extracellular and intracellular endosomal digestions and facilitate cytoplasmic delivery [43,44]. Figure 2 illustrates the different strategies and mechanisms that aim to modulate specific cytoplasmic delivery.

## 6. Nanocarriers in Drug Delivery

Mucoadhesive nanocarriers are commonly the more preferred form of drug delivery due to their adhesiveness property [45]. This bioadhesion that is offered may be one of three different types, namely adhesion between two biological components (e.g., the aggregation of platelets during the process of wound healing), adhesion of biological cells to an artificial substrate (e.g., the adhesion of experimental cells to culture wells), or adhesion of artificial substances to biological cells (e.g., the adhesion of hydrogels to biological cells). The third category of adhesion could also be referred to as mucoadhesion, where attachment of a drug-loaded carrier occurs to the mucous membrane [46]. There are a number of mechanisms that are proposed to be the source of mucoadhesion, of which the wetting mechanism is the oldest one. The adhesives form anchors after overcoming the surface tension between the surfaces and the penetration of the adhesive molecules into the irregular surface [47]. Alternatively, the diffusion mechanism describes the penetration of polymer chains into the glycoprotein mucin chains [48]. Other mechanisms include the electrostatic mechanism by exchange of electrons, the adsorption mechanism due to weak forces, and the fracture theory by separation of two surfaces after adhesion between the adhering surface and adhesive interface [46,49]. The oral mucoadhesive drug delivery systems, including sublingual and buccal delivery systems, have evoked great interest in drug development due to their convenience and accessibility [50].

There are several polymers that are available commercially which are widely incorporated in the pharmaceutical formulation that is used to obtain mucoadhesive properties (e.g., chitosan, poly(acrylic acid) or Carbopol, poly(vinyl pyrrolidone), poly(vinyl alcohol), poly(ethylene glycol), poly(hydroxyethyl methacrylate), etc.), where the degree of adhesion could be influenced by the hydrophilicity, crosslinking and swelling ability, molecular weight, spatial orientation, the concentration of the bioadhesive polymer as temperature changes, pH, and any additional influencing agent in the polymeric dispersion [46]. The mucous membrane in the buccal [51], ocular [52], intranasal [53], vaginal [54], and rectal [55] areas, etc., have already been explored in order to facilitate the prolonged residence of the mucoadhesive formulations at the site of delivery with moderated release pattern, which facilitates the transportation of the therapeutics to the diseased site for improved efficacy. With the concept of this mucoadhesion, a targeted delivery approach of therapeutics to oral cancer has also been made for improved efficacy. The following section of this article highlights the different strategies of targeted delivery of chemotherapeutics in the treatment of oral cancer with a special emphasis on the cytoplasmic delivery of anticancer agents using mucoadhesive-based nanoformulations.

### Multifunctional Properties of Nanocarriers

Mucoadhesive nanocarriers have a plethora of functions for initiating disease diagnosis and its treatment. Emerging mucoadhesive drug delivery methods for sustained pharmacological action are gaining attention due to mucosal localization and the controlled release of medicaments (APIs) [56]. Recently, low molecular weight heparin (LMWH) has been effective in the treatment of ulcerative colitis (UC). LMWH-loaded nanoparticles (trimethyl chitosan (TMC) NPs and sodium alginate [SA]-TMC-NPs) were prepared and evaluated in a series of studies, which included their stabilities, drug release, adhesion, permeation across mucosa, cytotoxic activity, anti-inflammatory, and anticoagulant activities, mucosal healing activity, biosafety, and ameliorative effects on experimental colitis. As a result, giving mice LMWH-loaded NPs orally for 5 days had considerable therapeutic effects, as seen by increased body weight, colon length, DAI score, MPO activity, and histological features. Furthermore, SA-TMC-NPs have a greater colon-targeting property than TMC-NPs, as evidenced by the reduced oral absorption (ATPP 38.95 s) and stronger mucoadhesion (kcps decreases 36.46%) to inflamed colon tissues. As a result, TMC-based NPs are effective oral colon-targeting drug delivery vehicles for LMWH, with potential for use in UC treatment [57]. Additionally, mucoadhesive nanocarriers emerged in the field of oral cancer diagnosis. Liposomes and their phospholipid manufacture have been frequently employed in cancer diagnosis research. Labeling with radionuclides like ^64^Cu, for example, is often accomplished by combining the radionuclide with an anchor molecule that is located inside the hydrophilic core or contained in the phospholipid bilayer. In comparison to ^18^F-FDG, Mahakian et al. claim that ^64^Cu liposomes can detect early cancers [58]. Not only liposomes, but dendrimers have also emerged as enticing technologies for detecting oral tumors. Wei et al. created DNA-dendrimer and polypyrrole (DDPpy) sensors in order to detect oral cancer biomarkers such as interleukin-8 RNA, interleukin-8 protein, and interleukin-1 protein, with improved specificity and affinity [59]. For targeted photoacoustic imaging, polymeric NPs can be employed as a contrast agent [60]. For the fluorescent endoscopic detection of oral cancers, a high-performance nanoparticle has been developed by Yang et al. By aiming at the folate receptors on oral cancer cells, folic-acid-conjugated chitosan NPs can improve nanoparticle endocytosis. By lowering the intensity between the chitosan and the drug, the N-succinyl chitosan (SCHI) polymer with a negative charge can promote 5-aminolevulinic acid (5-ALA) release in oral cancer cells [61]. Chitosan NPs can encapsulate ellagic acid [62], glycyrrhizic acid [63], and other anticancer medicines for topical and local delivery to oral cancers, preserving them against biological deactivation. As well as chitosan, other polymeric mucoadhesive nanoparticles have also shown excellent potential. Docetaxel-loaded poly(lactic-co-glycolic acid) (PLGA) NPs that are delivered locally to the tumor site exhibit improved antiproliferative efficiency [64].

## 7. Treatment Strategies to Enhance Targeted Delivery to Oral Cancer

Tissue alteration is a common phenomenon in oral cancer. As was discussed earlier, the available treatment options for oral dysplastic lesions are surgery, radiotherapy, and chemotherapy, used either alone or in a combination modality. This section focuses on the systemic or local delivery of anticancer drugs and the need for novel therapeutics to treat oral cancer effectively.

### 7.1. Systemic Delivery of Conventional and Novel Drug Delivery Systems

Conventional treatment in chemotherapy that is used for oral cancer treatment involves tablets, capsules, or parenteral delivery systems. Anticancer drugs are used alone or in combination for the treatment of oral cancer. However, the major setback for hydrophobic chemotherapeutics is their poor ability to negotiate non-specific tissue distribution, low solubility, permeability, and poor bioavailability [65]. In addition, intravenous or oral administration of highly cytotoxic drugs with non-specific tissue distribution causes significant damage to healthy tissues with critical adverse effects. A higher concentration of anticancer drugs in bodily fluid after oral administration is also a noticeable limitation [66]. Enhancement of the efficacy of the chemotherapeutics with lesser side effects could be achieved via a time-specific drug administration, as time plays a vital role in therapeutic efficacy and drug toxicity. Patients who have oral cancer suffered less from nausea, vomiting, and neutropenia when receiving evening Docetaxel, cisplatin plus fluorouracil (DCF) dosing than morning administration [67]. These promising results indicate the potential of chrono-chemotherapy as a novel strategy for oral cancer treatment.

**Figure 3:**
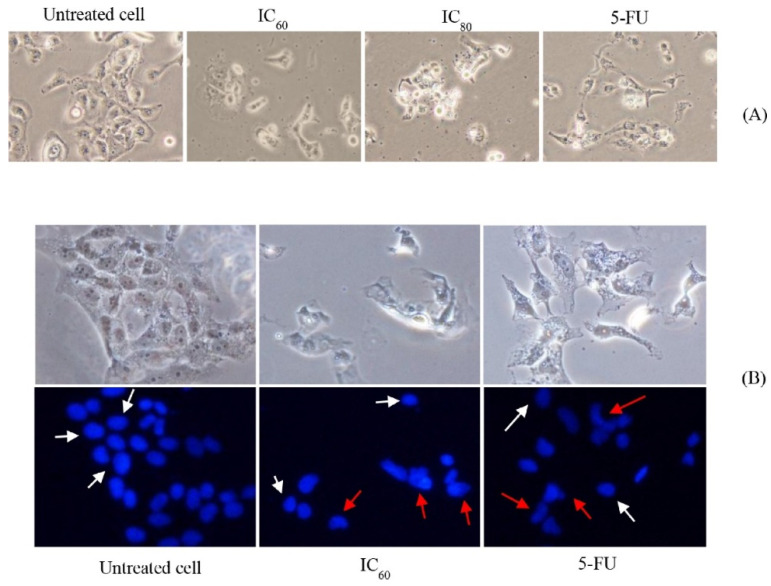
(**A**) Microscopic image of KON cancer cells exposed to NE and 5-FU. (**B**) Fluorescent images of KON cancer cells after treatment with control, NE (80:20) at IC60 concentration, and 5-FU; White and red arrows show normal nucleus and nucleus fragmentation, respectively [73].

Another approach to overcoming conventional drug delivery limitations is the development of targeted or advanced drug delivery systems using different polymers. This could improve patient compliance and drug efficiency. For instance, paclitaxel is widely used as a chemotherapeutic agent to treat cancer; however, its low solubility and permeability limit its therapeutic efficacy [68,69]. IV administration of paclitaxel, the usual delivery method, leads to severe side effects due to the distribution of the drug throughout the patient’s body, [70]. In this regard, Nakakaji et al. developed a magnetized conjugate which covalently linked N,N′-Bis(salicylidene)ethylenediamine iron (Fe(Salen)) to paclitaxel, (M-PTX). Their results showed that M-PTX improved the apoptosis of human oral cancer cell lines with marked contrast intensity observed in MRI. Furthermore, the accumulation of M-PTX after IV administration at the tumor site was facilitated by an external magnet. The anti-tumor effect was significantly higher compared to conventional IV administration of paclitaxel [71]. Similarly, spearmint oil contains terpene derivatives that have cytotoxicity against various tumor cells [72]. The application of spearmint oil in cancer treatment is limited due to its poor water solubility. In this regard, a nanoemulsion was produced via the phase inversion method and it showed excellent stability. Virgin coconut oil and polyoxyethylene castor oil derivatives were selected as the oil and surfactant, respectively, in the proportion of 80:20. The optimized nanoemulsion was evaluated for its cytotoxic effects and the mechanism of the cell death of KON cells. Figure 3 shows that, after treating the cells with the nanoemulsion, the cells’ shrinkage and loss of cell adhesion were similar to the results that were obtained after treating cells with 5-FU. The staining with DAPI probes showed nucleus fragmentation, which indicated that apoptosis was the cause of the cell death [73]. However, further studies are needed for the development of a clinical application.

### 7.2. Local Delivery of Conventional and Mucoadhesive Novel Drug Delivery Systems for Cytoplasmic Delivery

The local delivery of anticancer drugs is a field which is still being explored in order to reach the target site for significant pharmacological effects. In most cases, local or topical treatments are considered in premalignant oral lesions or patients who are at a high risk of recurrent cancer. The majority of the local formulations involve advanced drug delivery and have mucoadhesive properties. In the next section, various mucoadhesive nanocarriers with local delivery as an effective strategy to enhance the therapeutic effects of the anticancer drug in oral cancer are discussed.

As discussed earlier, mucoadhesion enhances the contact time to the mucous membrane and gives sufficient time for nanocarriers to penetrate and exert the targeted delivery. Amongst the different nanocarriers, NPs, liposomes, and nanoemulsions have been explored in order to deliver chemotherapeutics to the cytoplasm of the cancer cells using the mucoadhesive platform for improved efficacy. Summary of literation is given in Table 1.

#### 7.2.1. Mucoadhesive Nanoparticles in the Enhanced Treatment of Oral Cancer

**Figure 4:**
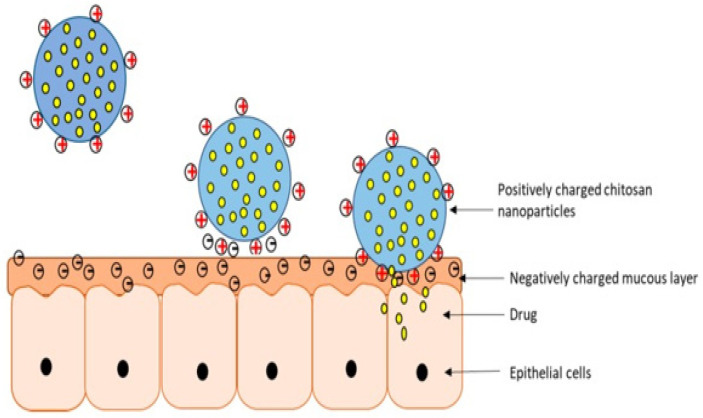
Mucoadhesive properties of chitosan-coated NPs allow prolonged drug delivery at the site of interest [75].

NPs are one of the carrier-based drug delivery systems that are currently presenting the ability to deliver oral cancer drugs [74]. NPs work by encapsulating the cytotoxic drugs, resulting in a controlled-release mechanism and preventing unwanted side effects such as systemic toxicity [74]. Meanwhile, mucoadhesive polymers such as chitosan, alginate, pectin, gelatin, etc., are being introduced into various drug delivery systems, including the buccal route, due to their advantages from adhering to the mucus membrane [75]. Remarkably, chitosan is a biopolymer containing easily modifiable chemical functional groups. Its role as a penetration enhancer facilitates the transcellular and paracellular transport of drugs [53,76]. These properties of chitosan enable tremendous potential in its therapeutic applications as a mucoadhesive polymer. With the various advantages of NPs and chitosan, chitosan-based NPs were introduced and received favorable outcomes from treating oral cancer due to their mucoadhesive nature and positive charge (Figure 4). Chitosan-based NPs can prolong drug release at the site of interest; thus the dosing frequency can be reduced [75].

Mazzarino et al., in their study, showed an interest in introducing curcumin into the mucoadhesive NPs in order to deliver curcumin locally for oral cancer [77]. The curcumin delivery was executed through the fabrication of polycaprolactone (PCL) NPs that were coated with chitosan polysaccharide, where chitosan was successfully proven to adsorb on the PCL surface. It was found to interact strongly with the glycoprotein mucin via electrostatic interactions, resulting in high curcumin concentration in the oral cavity [77]. The chitosan-coated curcumin-loaded PCL NPs significantly decreased the SCC-9 human oral cancer cells’ viability (45%) through apoptosis in cytotoxicity studies. However, free curcumin reduced the cell viability in a much higher percentage (90%), which might be due to the slower release of curcumin in the encapsulated core [77]. Despite the lower ability to reduce the cancer cell viability in similar doses of free curcumin and encapsulated curcumin, in vivo studies are encouraged to be carried out to determine the proper therapeutic amount that should be incorporated into NPs [77].

Alternatively, Matos et al. had explored the development of mucoadhesive chitosan NPs encapsulating oxaliplatin. The ex vivo penetration of NPs was investigated under both passive and iontophoretic treatments on porcine oral mucosa. Chitosan NPs entrapping oxaliplatin portrayed a small hydrodynamic size of less than 200 nm with a narrow size distribution and positive zeta potential [21]. These NPs have a biphasic release pattern consisting of a burst release followed by a sustained release. Besides these traits, chitosan NPs have been shown to increase the drug penetration at oral mucosa three-fold. This rate was constant even when the mucosa was “washed” with a buffer in order to mimic salivation. Moreover, chitosan NPs were found to increase the rate of cancer cells’ apoptosis [21]. The application of iontophoresis has been found to increase the amount of oxaliplatin which penetrated to the mucosa by two-fold. In short, topical therapy with chitosan NPs, enhanced by applying iontophoresis, is a potential approach to treat oral carcinoma or act as an adjuvant in treatments involving radiotherapy [21]. Similarly, to minimize the side effects of cytotoxic drugs and achieve a high concentration at the tumor site, catechol-modified chitosan/hyaluronic acid NPs (Cat-NPs) that were loaded with doxorubicin (DOX) were synthesized for oral cancer treatment. These NPs, of 160 nm in size, showed excellent mucoadhesion to oral mucosa with a sustained local cytotoxic drug delivery. The negative charge on the modified NPs demonstrated better mucoadhesion on ex vivo porcine oral mucosal tissues than unmodified NPs. The significant growth inhibition of the HN22 cell line with low half-maximal inhibitory concentration (IC50) was observed after treatment with DOX-loaded Cat-NPs. Additionally, more extensive cellular uptake, accumulation, and cancer cell apoptosis were observed by DOX-loaded Cat-NPs than free DOX. These findings reflect the potential of DOX-Cat-NPs for oral cancer treatment; however, in vivo and clinical studies are required to ensure their safety and efficacy [78]. Similarly, Wang et al. fabricated the nanosized PLGA/NR7 NP for cellular delivery of CDDP. Their results revealed that NPs have higher cellular uptake and excellent anticancer effects. This is attributed to targeting moiety NR7 which allows receptor-mediated internalization [79]. For cancer cell targeting and enhanced gastric permeability, a polymeric scaffold was originally created by grafting folic acid and thiol groups on chitosan (CS). Furthermore, by microwave irradiation, silver nanoclusters (Ag NCs) were generated in situ within the CS scaffold, core-shell nanocapsules (NCPs) were prepared with hydrophobic docetaxel (DTX) in the core, and Ag NCs were embedded in CS into the shell. When DTX and Ag NCs were delivered together, a considerable cytotoxicity synergism (300 times) was seen against breast cancer MDA-MB-231 cells. In comparison to the control DTX suspension, the DTX-Ag-NCPs increased their bioavailability after oral administration due to improved drug transport across the gut (9 times), circulation half-life (6.7 times), and mean residence duration (6.7 times). Furthermore, a 14-day acute oral toxicity study of the DTX-Ag-NCPs in mice was conducted, with no significant evidence of toxicity found in blood biochemistry parameters, organ to body weight index, or histopathology of liver and kidney tissues. This suggests that the DTX-Ag-NCPs are safe and effective as a hybrid nanocarrier for the biocompatible delivery of metal nanoclusters [80].

On the other hand, for the OSCC therapy, researchers developed a combinational chemo-photothermal therapy using vincristine (VCR) as a phytochemical anticancer agent and plasmonic gold nanorods (GNRs) as a photothermal reagent (Darwish et al., 2020). VCR was physically encased within the polymeric corona via chem-covalent assembly around silica-coated gold nanorods, which was based on the self-assembly of amphiphilic poly (*d,l*-lactide-co-glycolide) (PLGA)-PEG polymers (GNRs). Under acidic intracellular circumstances, the rupture of amide connections induced long-term VCR release, revealing the generated combinational therapeutic nanoprobes as viable candidates for clinical translation [81].

Sayed et al. described the development of anti-epithelial growth factor receptor (EGFR) antibody-conjugated Au NPs for the treatment of OSCC. In vitro experiments revealed that OSCC cells did not require high levels of energy in order to generate the photothermal destruction of anti-EGFR/Au conjugates and the clinical findings have shown that near-infrared (NIR) laser light could enable the effective delivery of anti-EGFR/Au conjugates into malignant cells with deep penetration [82].

Overall, it can be concluded that the mucoadhesive NPs could be a useful platform for treating oral cancer by improving therapeutic activity and drug availability at the site of action.

#### 7.2.2. Mucoadhesive Liposomes for the Treatment of Oral Cancer

**Figure 5:**
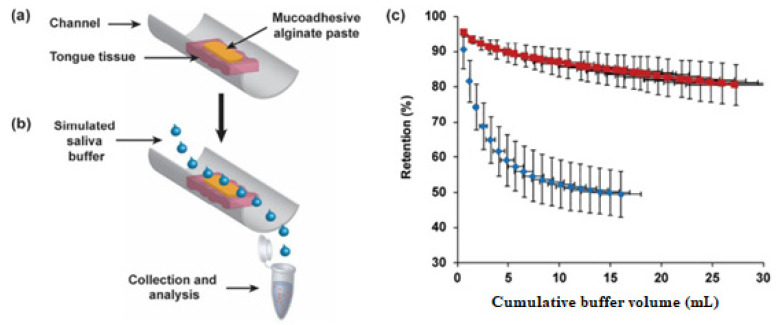
Assessment of mucoadhesion. (**a**,**b**)–preparation and buffer flow on sample, (**c**) Mucoadhesion evaluation of 3% (*w*/*v*) alginate-fluorescein (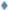) paste; (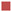) cross-linked paste on porcine tongue. Reprinted from ref. [88] with permission from Elsevier (2018).

Liposomes are concentric vesicles in which an aqueous core is enclosed by one or more phospholipid bilayers [83,84,85]. Most clinically approved liposomes range from the size of 50–300 nm, where the hydrophilic and hydrophobic drug molecules can be entrapped in the aqueous core and lipid bilayers, respectively [83,84]. In recent years, liposomes have been under extensive investigation as drug carriers for cytotoxic drugs due to their proven ability to enhance drug absorption at a localized site and regulate the release rate of any incorporated drugs [86]. In this regard, Jin et al. formulated a targeted methotrexate-entrapped liposomal mucoadhesive buccal film (M-LP-F7) for oral cancer treatment. The optimized methotrexate-loaded liposomes (M-LP) had a diameter range of 137.4 ± 2.6 nm and zeta-potential of 36.0 ± 3.1 mV, with entrapment efficiency of 73.4 ± 1.7% [87]. These liposomes were cast in an optimized mucoadhesive buccal film (F7), which was composed of chitosan, HPMC, and PVA. The physicochemical properties, in vitro release profile, and cell-line cytotoxicity of the developed formulation were evaluated. The film was soft, flexible, malleable, and had good aesthetic properties [87]. The in vitro release study of M-LP-F7 demonstrated a sustained release profile for 6 h that was attributed to chitosan, HPMC, and PVA. This ability was attributed to the mucoadhesive swellable film matrix which was able to control the drug release rate [87]. Notably, chitosan strengthened the polymer network and maintained the film’s integrity and liposomal vesicles. The cytotoxicity of M-LP-F7 on the human oral squamous carcinoma cell line (HSC-3 cells) via MTT assay showed a marked reduction in IC50, potentially contributed to by the enhanced methotrexate permeation in the form of the liposome. Compared to methotrexate alone, the developed formulation had caused a 3-fold increment of the percentage of apoptotic cells, which were associated with the underlying mitochondrial membrane potential disruption, pro-oxidant effect, and reactive oxygen species accumulation. Thus, the methotrexate-entrapped liposome-laden mucoadhesive film had shown suitable characteristics in delivering site-specific, prolonged treatment for oral cancer at lower drug doses, thereby suppressing systemic toxicity compared to other routes of administration [87]. Another study by Shtenberg et al. focused on developing doxorubicin-loaded liposomes in a mucoadhesive cross-linked alginate oral paste for the localized treatment of oral cancer. The liposomes’ size distribution ranged from 122–137 nm [88]. The size and structural morphology of the liposomes were stable and maintained throughout the experimental conditions. The superior mucoadhesive property of alginate was evidenced by the 80% retention rate of the formulated paste on porcine tongue tissue after continuous elution with a buffer. Alginate has been known for its ability to create hydrogen bonds with mucin-type glycoprotein, resulting in excellent mucoadhesive properties (Figure 5) [88]. On the other hand, another major advantage of alginate is its ion-responsive property. The cross-linking of alginate in the presence of positively charged ions, such as calcium ions, controls the drug’s release rate over more than 8 h. The cytotoxicity of the developed formulation was examined on the human tongue squamous cell carcinoma cell line (CAL-27) via an MTT assay. Similarly, the cytotoxicity results significantly reduced the cell viability levels to 38% and 15% after 24 and 48 h, respectively. Hence, the authors concluded that this innovative formulation, allowing both desired mucoadhesive characteristics and sustained release of doxorubicin upon lingual administration, could be a new potential treatment for oral cancer [88].

#### 7.2.3. Mucoadhesive Nanoemulsion in the Enhanced Treatment of Oral Cancer

Nanoemulsions are isotropic dispersion systems that comprise two immiscible liquids that are stabilized by surfactants with a droplet size of 20 nm to 600 nm [89,90,91]. Nanoemulsions are efficient controlled drug delivery systems because they increase the half-life and bioavailability of the drug at the specific tumor site for improved therapeutic activity with simultaneously decreased drug toxicity [92]. Thus, this novel delivery method has been widely explored as a carrier for cancer treatment. Srivastava et al. had developed a combination of 5-fluorouracil (5-Fu) and curcumin (Cur)-loaded nanoemulsion (5-Fu-Cur-NE) in order to increase the anticancer potency against OSCC [92]; 5-Fu-Cur-NE has a mean droplet size of 150 nm to 200 nm and a zeta potential of −25.70 mV to −37.91 mV. During this aforementioned in vitro release study, which took place over four days, 5-Fu-Cur-NE exhibited higher release in an acidic pH environment (6.7–7.6) with acceptable stability than in an alkaline pH environment. A dose-dependent anticancer effect with lower IC50 value was observed in a cytotoxicity study on OSCC cells (SCC090 and SCC 152). Besides this, it showed a synergistic anticancer effect in killing cancer cells. A maximum intracellular uptake of 5-Fu-Cur-NE in cancer cells can cause a change in their protein expression, leading to cell apoptosis [92]. On the other hand, the anticancer drug genistein (Gen) is a promising candidate for oral cancer treatment; however, its poor solubility and high first-pass metabolism limit its clinical application. Incorporating Gen-nanoemulsion with a chitosan coating enhances mucoadhesive properties, improving its oral and transdermal bioavailability in lozenge/buccal tablets [19]. Chitosan-coated Gen-nanoemulsion manifests good stability with the mean size of 120 nm to 140 nm and zeta-potential between +11.9 mV to +20.5 mV [19]. Gavin et al. compared chitosan-coated and aqueous coated Gen-containing NE formulations and showed activity against in vitro cytotoxicity test on human pharyngeal squamous cell carcinoma (FaDu). Nevertheless, the chitosan-coated Gen-containing nanoemulsions exhibited stronger cytotoxic activity after 48 h of incubation with the cancer cells. Despite their slow-release profile (<36 h compared with aqueous Gen-nanoemulsion), they had the advantage of killing cancer cells by releasing the drugs in juxtaposition. After processing NE into buccal tablet form, the effects were equivalent to those of a nanoemulsion with substantial anticancer efficiency against oropharyngeal carcinomas [19].

**Figure 6:**
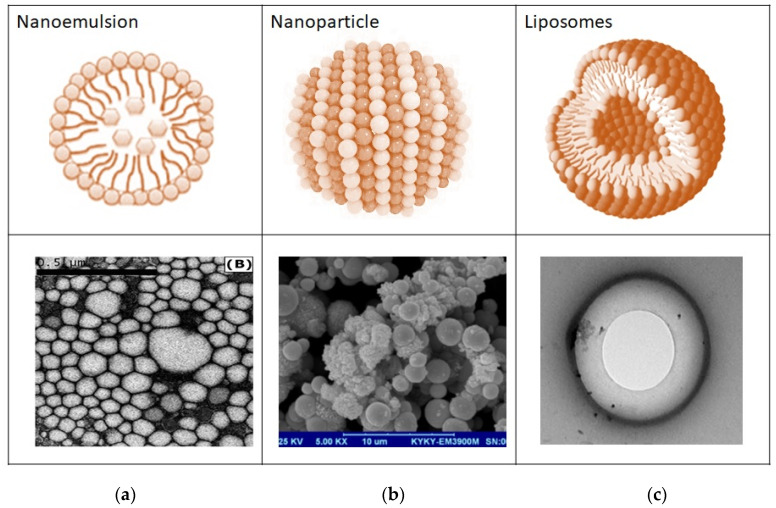
Electron microscopy analysis of (**a**) nano emulsion (reprinted from ref. [93] with permission from Elsevier, 2012), (**b**) nanoparticle [94], and (**c**) liposomes [95].

Figure 6 further explains the structural differences of nanocarriers based on their SEM images. The TEM micrographs demonstrate that most liposomes have a diameter in the nano range and have two compartments. Furthermore, the electron microscopy analysis of nanoemulsions confirmed that the oil droplets were spherical in shape and uniformly distributed within the nanoemulsions. The SEM morphology of the nanoparticles shows their spherical shape and rigid structure.

**Table 1:** Anti-cancer drug-loaded mucoadhesive nanocarriers for the treatment of oral carcinoma.

Types of Nanocarriers	Composition of Nanoparticles	Anticancer Drug	Animal/Ex Vivo/Cell Lines	Outcome	References
Nanoparticles	Polycaprolactone chitosan	Curcumin	Porcine esophagi	Chitosan-based NP was found to interact strongly with glycoprotein mucin in the oral cavity through electrostatic interactions In vitro studies demonstrated that the curcumin-loaded PCL NPs that were coated with chitosan decreased the viability of SCC-9 human oral cancer cells significantly by inducing apoptosis	[77]
	Chitosan	Oxaliplatin	Porcine mucosa	Chitosan NPs increased three-fold the drug’s penetration, provided a ‘burst effect’ upon the drug release followed by a longer-term drug penetration, and increased the rate of cells that entered apoptosis Iontophoresis doubled the amount of OXPt that was transported to the mucosa	[21]
	Chitosan, hyluronic acid	Doxurubicin	Porcine oral mucosal tissues HN22 cell lines	Significant IC_50_ reduction High mucoadhesion to oral mucosa Sustained release Higher cellular uptake and cytotoxicity compared to a free drug	[78]
Liposomes	Chitosan, HPMC, and PVA buccal film	Methotrexate	Human oral squamous carcinoma cell line (HSC-3 cells)	Significant IC_50_ reduction 3-fold increment of the percentage of apoptotic cells Sustained drug release for 6 h	[87]
	Alginate oral paste	Doxorubicin	Human tongue squamous cell carcinoma cell line (CAL-27)	Significant reduction in cell viability to 38% and 15% after 24 and 48 h, respectively Prolonged drug release for 8 h	[88]
Nanoemulsion	Tween 80 and soya oil, glycerol, water	5-fluorouracil & curcumin	Oral squamous cells carcinoma (SCC090 and SCC 152)	5-Fu-Cur-NE exhibited greater antitumor activity in an acidic pH environment (6.7–7.6) 5-fluorouracil and curcumin exhibited a synergistic anticancer effect Changed protein expression, leading to cell apoptosis Reduced IC_50_ value to approximately 28.05%	[92]
	Chia seed oil and α-tocopherol, TPGS, MCC, dextrose	Genistein	FaDu human pharyngeal squamous cell carcinoma	Chitosan-coated Gen-containing NE exhibited a more potent cytotoxic activity than aqueous-coated Gen-containing NE Potential application as a maintenance therapy for a patient who is waiting for surgical removal	[19]

## 8. Conclusions

With diverse nanotechnological techniques for oral cancer therapy, significant challenges and advancements have been identified. These carriers can be loaded with anticancer cargoes in order to target malignant cells with great effectiveness and less damage to healthy cells, showing a site-specific delivery behavior and based on these targeted drug delivery systems with tailored architectures and diverse physicochemical features. In this systematic review, polymeric nanoparticles, inorganic nanoparticles, liposomes, nano-lipids, hydrogels, and numerous biomimetic modes of drug administration have been thoroughly investigated as therapeutic possibilities for the treatment of oral cancers. Most of these carriers demonstrated a tremendous potential as alternatives that may be used in order to overcome the restrictions that are associated with oral medicines and conventional formulations by harnessing their delicate framework correlations. Nonetheless, with the present targeted drug delivery systems, only a few examples of rigorous clinical research have been conducted thus far, revealing that improving clinical efficiency, controlling the drug’s release, and reducing side effects are extremely difficult. One of the most significant challenges for commercialization is the relatively complex architectures of most drug carriers, which results in serious issues such as their time-consuming and costly manufacturing. Despite the diverse cellular mechanisms that are operating in the OSCC context, high-loading drug dosages and optimum drug release patterns for these systems for oral cancer therapy remain a primary priority. Clinical trials are another major issue that must be addressed in all kinds of cancer, including oral cancer. Currently, the majority of studies are conducted in vitro or in vivo. By involving biomedical engineers, cancer biologists, medical specialists, and healthcare professionals, continued research regarding the mucoadhesive nanocarrier should be conducted in order to further investigate its effectiveness on OSCCs.

## Data Availability

The data are freely available.

## References

[1] Irani S. (2020). New insights into oral cancer—Risk factors and prevention: A review of literature. Int. J. Prev. Med..

[2] WHO Oral Health https://www.who.int/news-room/fact-sheets/detail/oral-health.

[3] Manikandan M., Rao A.K.D.M., Arunkumar G., Manickavasagam M., Rajkumar K.S., Rajaraman R., Munirajan A.K. (2016). Oral squamous cell carcinoma: MicroRNA expression profiling and integrative analyses for elucidation of tumourigenesis mechanism. Mol. Cancer.

[4] Bray F., Ferlay J., Soerjomataram I., Siegel R.L., Torre L.A., Jemal A. (2018). Global cancer statistics 2018: GLOBOCAN estimates of incidence and mortality worldwide for 36 cancers in 185 countries. CA Cancer J. Clin..

[5] Nör J., Gutkind J. (2018). Head and Neck Cancer in the New Era of Precision Medicine. J. Dent. Res..

[6] Chi A.C., Day T.A., Neville B.W. (2015). Oral cavity and oropharyngeal squamous cell carcinoma-an update. CA Cancer J. Clin..

[7] Li H., Shi L., Wei J., Zhang C., Zhou Z., Wu L., Liu W. (2016). Cellular uptake and anticancer activity of salvianolic acid B phospholipid complex loaded nanoparticles in head and neck cancer and precancer cells. Colloids Surf. B Biointerfaces.

[8] Jiang X., Wu J., Wang J., Huang R. (2019). Tobacco and oral squamous cell carcinoma: A review of carcinogenic pathways. Tob. Induc. Dis..

[9] Dhanuthai K., Rojanawatsirivej S., Thosaporn W., Kintarak S., Subarnbhesaj A., Darling M., Kryshtalskyj E., Chiang C.-P., Shin H.-I., Choi S.-Y. (2018). Oral cancer: A multicenter study. Med. Oral Patol. Oral Cir. Bucal.

[10] Rivera C., Venegas B. (2014). Histological and molecular aspects of oral squamous cell carcinoma (Review). Oncol. Lett..

[11] Kushima M., Tsuda Y., Morishita A., Fukuda M., Akita H., Ichihara M., Morioka M. (2012). Koilocytosis. J. Jpn. Soc. Clin. Cytol..

[12] Marcazzan S., Varoni E.M., Blanco E., Lodi G., Ferrari M. (2018). Nanomedicine, an emerging therapeutic strategy for oral cancer therapy. Oral Oncol..

[13] Jain A., Madu C.O., Lu Y. (2021). Phytochemicals in Chemoprevention: A Cost-Effective Complementary Approach. J. Cancer.

[14] Azharuddin M., Roberg K., Dhara A.K., Jain M.V., D’Arcy P., Hinkula J., Slater N.K.H., Patra H.K. (2019). Dissecting multi drug resistance in head and neck cancer cells using multicellular tumor spheroids. Sci. Rep..

[15] Blanco E., Shen H., Ferrari M. (2015). Principles of nanoparticle design for overcoming biological barriers to drug delivery. Nat. Biotechnol..

[16] Wong T., Wiesenfeld D. (2018). Oral Cancer. Aust. Dent. J..

[17] Gomathi M., Ayisha Hamna T.P., Jijo A.J., Saradha Devi K.M., Arul N., Balachandar V. (2019). Recent advances in radiotherapy and its associated side effects in cancer—A review. J. Basic Appl. Zool..

[18] Mariadoss A.V.A., Vinayagam R., Xu B., Venkatachalam K., Sankaran V., Vijayakumar S., Bakthavatsalam S.R., Mohamed S.A., David E. (2019). Phloretin loaded chitosan nanoparticles enhance the antioxidants and apoptotic mechanisms in DMBA induced experimental carcinogenesis. Chem. Interact..

[19] Elbayoumi T., Gavin A., Pham J., Wang D., Brownlow B. (2015). Layered nanoemulsions as mucoadhesive buccal systems for controlled delivery of oral cancer therapeutics. Int. J. Nanomed..

[20] Gómez-Guillén M.C., Montero M.P. (2021). Enhancement of oral bioavailability of natural compounds and probiotics by mucoadhesive tailored biopolymer-based nanoparticles: A review. Food Hydrocoll..

[21] Matos B.N., Pereira M.N., Bravo M.D.O., Filho M.C., Saldanha-Araújo F., Gratieri T., Gelfuso G.M. (2019). Chitosan nanoparticles loading oxaliplatin as a mucoadhesive topical treatment of oral tumors: Iontophoresis further enhances drug delivery ex vivo. Int. J. Biol. Macromol..

[22] Sankar V., Hearnden V., Hull K., Juras D.V., Greenberg M.S., Kerr A.R., Lockhart P.B., Patton L., Porter S., Thornhill M. (2011). Local drug delivery for oral mucosal diseases: Challenges and opportunities. Oral Dis..

[23] Kurian Mathew A. (2015). Oral local drug delivery: An overview. Pharm. Pharmacol. Res..

[24] Sohi H., Ahuja A., Ahmad F.J., Khar R.K. (2010). Critical evaluation of permeation enhancers for oral mucosal drug de-livery. Drug Dev. Ind. Pharm..

[25] Macedo A., Castro P.M., Roque L., Thomé N.G., Reis C., Pintado M.M., Fonte P. (2020). Novel and revisited approaches in nanoparticle systems for buccal drug delivery. J. Control. Release.

[26] Nguyen S., Hiorth M. (2015). Advanced drug delivery systems for local treatment of the oral cavity. Ther. Deliv..

[27] Roncato F., Rruga F., Porcù E., Casarin E., Ronca R., Maccarinelli F., Realdon N., Basso G., Alon R., Viola G. (2018). Improvement and extension of anti-EGFR targeting in breast cancer therapy by integration with the Avidin-Nucleic-Acid-Nano-Assemblies. Nat. Commun..

[28] Johnson D.E., Burtness B., Leemans C.R., Lui V.W.Y., Bauman J.E., Grandis J.R. (2020). Head and neck squamous cell carcinoma. Nat. Rev. Dis. Primers.

[29] Kato M.G., Baek C.-H., Chaturvedi P., Gallagher R., Kowalski L.P., Leemans C.R., Warnakulasuriya S., Nguyen S.A., Day T.A. (2020). Update on oral and oropharyngeal cancer staging–International perspectives. World J. Otorhinolaryngol. Head Neck Surg..

[30] Calixto G., Fonseca-Santos B., Chorilli M., Bernegossi J. (2014). Nanotechnology-based drug delivery systems for treatment of oral cancer: A review. Int. J. Nanomed..

[31] Ketabat F., Pundir M., Mohabatpour F., Lobanova L., Koutsopoulos S., Hadjiiski L., Chen X., Papagerakis P., Papagerakis S. (2019). Controlled Drug Delivery Systems for Oral Cancer Treatment—Current Status and Future Perspectives. Pharmaceutics.

[32] Yao Y., Zhou Y., Liu L., Xu Y., Chen Q., Wang Y., Wu S., Deng Y., Zhang J., Shao A. (2020). Nanoparticle-Based Drug Delivery in Cancer Therapy and Its Role in Overcoming Drug Resistance. Front. Mol. Biosci..

[33] Chen X.J., Zhang X.Q., Liu Q., Zhang J., Zhou G. (2018). Nanotechnology: A promising method for oral cancer detection and diagnosis. J. Nanobiotechnology.

[34] Gao A., Teng Y., Seyiti P., Yen Y., Qian H., Xie C., Li R., Lin Z. (2019). Using Omniscan-Loaded Nanoparticles as a Tumor-Targeted MRI Contrast Agent in Oral Squamous Cell Carcinoma by Gelatinase-Stimuli Strategy. Nanoscale Res. Lett..

[35] Kim C.S., Ingato D., Wilder-Smith P., Chen Z., Kwon Y.J. (2018). Stimuli-disassembling gold nanoclusters for diagnosis of early stage oral cancer by optical coherence tomography. Nano Converg..

[36] Yadav P., Jain J., Sherje A.P. (2021). Recent advances in nanocarriers-based drug delivery for cancer therapeutics: A review. React. Funct. Polym..

[37] Reinholz J., Landfester K., Mailänder V. (2018). The challenges of oral drug delivery via nanocarriers. Drug Deliv..

[38] Hua S. (2019). Advances in Nanoparticulate Drug Delivery Approaches for Sublingual and Buccal Administration. Front. Pharmacol..

[39] Sivashankari P.R., Prabaharan M. (2017). Chitosan/Carbon-Based Nanomaterials as Scaffolds for Tissue Engineering.

[40] Gao Z., Zhang L., Sun Y. (2012). Nanotechnology applied to overcome tumor drug resistance. J. Control. Release.

[41] Pathak C., Vaidya F.U., Pandey S.M. (2019). Mechanism for Development of Nanobased Drug Delivery System. Applications of Targeted Nano Drugs and Delivery Systems.

[42] Caro C., Pozo D. (2015). Polysaccharide Colloids as Smart Vehicles in Cancer Therapy. Curr. Pharm. Des..

[43] Battistella C., Klok H.-A. (2017). Controlling and Monitoring Intracellular Delivery of Anticancer Polymer Nanomedicines. Macromol. Biosci..

[44] Parodi A., Corbo C., Cevenini A., Molinaro R., Palomba R., Pandolfi L., Agostini M., Salvatore F., Tasciotti E. (2015). Enabling cytoplasmic delivery and organelle targeting by surface modification of nanocarriers. Nanomedicine.

[45] De Felice F., Cavallini C., Barlattani A., Tombolini M., Brugnoletti O., Tombolini V., Polimeni A. (2019). Nanotechnology in Oral Cavity Carcinoma: Recent Trends and Treatment Opportunities. Nanomaterials.

[46] Shaikh R., Raj Singh T., Garland M., Woolfson A., Donnelly R. (2011). Mucoadhesive drug delivery systems. J. Pharm. Bioallied Sci..

[47] McBain J.W., Hopkins D.G. (1925). On Adhesives and Adhesive Action. J. Phys. Chem..

[48] Jiménez-Castellanos M.R., Zia H., Rhodes C.T. (1993). Mucoadhesive Drug Delivery Systems. Drug Dev. Ind. Pharm..

[49] Wakaskar R.R. (2017). Role of Nanoparticles in Drug Delivery Encompassing Cancer Therapeutics. Int. J. Drug Dev. Res..

[50] Gupta S., Das S., Singh A., Ghosh S. (2021). A Brief Review on Bucco-adhesive Drug Delivery System. J. Drug Deliv. Ther..

[51] Alkhalidi H.M., Hosny K.M., Rizg W.Y. (2020). Oral Gel Loaded by Fluconazole-Sesame Oil Nanotransfersomes: Development, Optimization, and Assessment of Antifungal Activity. Pharmaceutics.

[52] Pandey M., Choudhury H., Aziz A.B.A., Bhattamisra S., Gorain B., Su J., Tan C., Chin W., Yip K. (2021). Potential of Stimuli-Responsive In Situ Gel System for Sustained Ocular Drug Delivery: Recent Progress and Contemporary Research. Polymers.

[53] Chin L.Y., Tan J.Y.P., Choudhury H., Pandey M., Sisinthy S.P., Gorain B. (2021). Development and optimization of chitosan coated nanoemulgel of telmisartan for intranasal delivery: A comparative study. J. Drug Deliv. Sci. Technol..

[54] Pandey M., Choudhury H., Abdul-Aziz A., Bhattamisra S.K., Gorain B., Carine T., Wee Toong T., Yi N.J., Win Yi L. (2020). Promising Drug Delivery Approaches to Treat Microbial Infections in the Vagina: A Recent Update. Polymers.

[55] El-Leithy E.S., Shaker D.S., Ghorab M.K., Abdel-Rashid R.S. (2010). Evaluation of Mucoadhesive Hydrogels Loaded with Diclofenac Sodium–Chitosan Microspheres for Rectal Administration. Aaps Pharmscitech.

[56] Yaqoob A.A., Ahmad H., Parveen T., Ahmad A., Oves M., Ismail I.M.I., Qari H.A., Umar K., Ibrahim M.N.M. (2020). Recent Advances in Metal Decorated Nanomaterials and Their Various Biological Applications: A Review. Front. Chem..

[57] Yan Y., Sun Y., Wang P., Zhang R., Huo C., Gao T., Song C., Xing J., Dong Y. (2020). Mucoadhesive nanoparticles-based oral drug delivery systems enhance ameliorative effects of low molecular weight heparin on experimental colitis. Carbohydr. Polym..

[58] Mahakian L.M., Farwell D.G., Zhang H., Seo J.W., Poirier B., Tinling S.P., Afify A.M., Haynam E.M., Shaye D., Ferrara K.W. (2014). Comparison of PET Imaging with 64Cu-Liposomes and 18F-FDG in the 7,12-Dimethylbenz[a]anthracene (DMBA)-Induced Hamster Buccal Pouch Model of Oral Dysplasia and Squamous Cell Carcinoma. Mol. Imaging Biol..

[59] Wei F., Liao W., Xu Z., Yang Y., Wong D.T., Ho C.-M. (2009). Bio/Abiotic Interface Constructed from Nanoscale DNA Dendrimer and Conducting Polymer for Ultrasensitive Biomolecular Diagnosis. Small.

[60] Xiong J., Feng J., Qiu L., Gao Z., Li P., Pang L., Zhang Z. (2019). SDF-1-loaded PLGA nanoparticles for the targeted photoacoustic imaging and photothermal therapy of metastatic lymph nodes in tongue squamous cell carcinoma. Int. J. Pharm..

[61] Yang S.-J., Lin C.-F., Kuo M.-L., Tan C.-T. (2013). Photodynamic Detection of Oral Cancers with High-Performance Chitosan-Based Nanoparticles. Biomacromolecules.

[62] Arulmozhi V., Pandian K., Mirunalini S. (2013). Ellagic acid encapsulated chitosan nanoparticles for drug delivery system in human oral cancer cell line (KB). Colloids Surf. B Biointerfaces.

[63] Cacciotti I., Chronopoulou L., Palocci C., Amalfitano A., Cantiani M., Cordaro M., Lajolo C., Callà C., Boninsegna A., Lucchetti D. (2018). Controlled release of 18-β-glycyrrhetic acid by nanodelivery systems increases cytotoxicity on oral carcinoma cell line. Nanotechnology.

[64] Gupta P., Singh M., Kumar R., Belz J., Shanker R., Dwivedi P.D., Sridhar S., Singh S.P. (2018). Synthesis and in vitro studies of PLGA-DTX nanoconjugate as potential drug delivery vehicle for oral cancer. Int. J. Nanomed..

[65] Hejmady S., Pradhan R., Alexander A., Agrawal M., Singhvi G., Gorain B., Tiwari S., Kesharwani P., Dubey S.K. (2020). Recent advances in targeted nanomedicine as promising antitumor therapeutics. Drug Discov. Today.

[66] Pandey M., Choudhury H., Yeun O.C., Yin H.M., Lynn T.W., Tine C.L., Wi N.S., Yen K.C., Phing C.S., Kesharwani P. (2018). Perspectives of Nanoemulsion Strategies in The Improvement of Oral, Parenteral and Transdermal Chemotherapy. Curr. Pharm. Biotechnol..

[67] Tsuchiya Y., Ushijima K., Noguchi T., Okada N., Hayasaka J.-I., Jinbu Y., Ando H., Mori Y., Kusama M., Fujimura A. (2018). Influence of a dosing-time on toxicities induced by docetaxel, cisplatin and 5-fluorouracil in patients with oral squamous cell carcinoma; a cross-over pilot study. Chronobiol. Int..

[68] Choudhury H., Gorain B., Karmakar S., Biswas E., Dey G., Barik R., Mandal M., Pal T.K. (2014). Improvement of cellular uptake, in vitro antitumor activity and sustained release profile with increased bioavailability from a nanoemulsion platform. Int. J. Pharm..

[69] Gorain B., Choudhury H., Pandey M., Kesharwani P. (2018). Paclitaxel loaded vitamin E-TPGS nanoparticles for cancer therapy. Mater. Sci. Eng. C.

[70] Chou P.-L., Huang Y.-P., Cheng M.-H., Rau K.-M., Fang Y.-P. (2020). Improvement of Paclitaxel-Associated Adverse Reactions (ADRs) via the Use of Nano-Based Drug Delivery Systems: A Systematic Review and Network Meta-Analysis. Int. J. Nanomed..

[71] Nakakaji R., Umemura M., Mitsudo K., Kim J.-H., Hoshino Y., Sato I., Masuda T., Yamamoto M., Kioi M., Koizumi T. (2018). Treatment of oral cancer using magnetized paclitaxel. Oncotarget.

[72] Jaafari A., Tilaoui M., Mouse H.A., M’Bark L.A., Aboufatima R., Chait A., Lepoivre M., Zyad A. (2012). Comparative study of the antitumor effect of natural monoterpenes: Relationship to cell cycle analysis. Rev. Bras. Farm..

[73] Tubtimsri S., Limmatvapirat C., Limsirichaikul S., Akkaramongkolporn P., Inoue Y., Limmatvapirat S. (2018). Fabrication and characterization of spearmint oil loaded nanoemulsions as cytotoxic agents against oral cancer cell. Asian J. Pharm. Sci..

[74] Ramalingam K., Poonia M., Goyal S., Sidhu S.K. (2017). Nanotechnology in oral cancer: A comprehensive review. J. Oral Maxillofac. Pathol..

[75] Mohammed M.A., Syeda J.T.M., Wasan K.M., Wasan E.K. (2017). An Overview of Chitosan Nanoparticles and Its Application in Non-Parenteral Drug Delivery. Pharmaceutics.

[76] Garg U., Chauhan S., Nagaich U., Jain N. (2019). Current Advances in Chitosan Nanoparticles Based Drug Delivery and Targeting. Adv. Pharm. Bull..

[77] Mazzarino L., Loch-Neckel G., Bubniak L.D.S., Mazzucco S., Santos-Silva M.C., Borsali R., Lemos-Senna E. (2015). Curcumin-Loaded Chitosan-Coated Nanoparticles as a New Approach for the Local Treatment of Oral Cavity Cancer. J. Nanosci. Nanotechnol..

[78] Pornpitchanarong C., Rojanarata T., Opanasopit P., Ngawhirunpat T., Patrojanasophon P. (2020). Catechol-modified chitosan/hyaluronic acid nanoparticles as a new avenue for local delivery of doxorubicin to oral cancer cells. Colloids Surf. B Biointerfaces.

[79] Wang Z.-Q., Liu K., Huo Z.-J., Li X.-C., Wang M., Liu P., Pang B., Wang S.-J. (2015). A cell-targeted chemotherapeutic nanomedicine strategy for oral squamous cell carcinoma therapy. J. Nanobiotechnology.

[80] Sohail M.F., Hussain S.Z., Saeed H., Javed I., Sarwar H.S., Nadhman A., Huma Z.-E., Rehman M., Jahan S., Hussain I. (2018). Polymeric nanocapsules embedded with ultra-small silver nanoclusters for synergistic pharmacology and improved oral delivery of Docetaxel. Sci. Rep..

[81] Darwish W.M., Abdoon A.S., Shata M.S., Elmansy M. (2020). Vincristine-loaded polymeric corona around gold nanorods for combination (chemo-photothermal) therapy of oral squamous carcinoma. React. Funct. Polym..

[82] El-Sayed I.H., Huang X., El-Sayed M.A. (2006). Selective laser photo-thermal therapy of epithelial carcinoma using anti-EGFR antibody conjugated gold nanoparticles. Cancer Lett..

[83] Karami N., Moghimipour E., Salimi A. (2018). Liposomes as a novel drug delivery system: Fundamental and pharmaceutical ap-plication. Asian J. Pharm..

[84] Sah A.K., Vyas A., Suresh P.K., Gidwani B. (2018). Application of nanocarrier-based drug delivery system in treatment of oral cancer. Artif. Cells Nanomed. Biotechnol..

[85] Gorain B., Al-Dhubiab B.E., Nair A., Kesharwani P., Pandey M., Choudhury H. (2021). Multivesicular Liposome: A Lipid-based Drug Delivery System for Efficient Drug Delivery. Curr. Pharm. Des..

[86] Erjavec V., Pavlica Z., Sentjurc M., Petelin M. (2006). In vivo study of liposomes as drug carriers to oral mucosa using EPR oximetry. Int. J. Pharm..

[87] Jin B., Dong X., Xu X., Zhang F. (2017). Development and inï¿½vitro evaluation of mucoadhesive patches of methotrexate for targeted delivery in oral cancer. Oncol. Lett..

[88] Shtenberg Y., Goldfeder M., Prinz H., Shainsky J., Ghantous Y., Abu El-Naaj I., Schroeder A., Bianco-Peled H. (2018). Mucoadhesive alginate pastes with embedded liposomes for local oral drug delivery. Int. J. Biol. Macromol..

[89] Jaiswal M., Dudhe R., Sharma P.K. (2015). Nanoemulsion: An advanced mode of drug delivery system. 3 Biotech.

[90] Chavda V.P. (2018). Nanobased Nano Drug Delivery. Applications of Targeted Nano Drugs and Delivery Systems.

[91] Choudhury H., Gorain B., Chatterjee B., Mandal U.K., Sengupta P., Tekade R.K. (2016). Pharmacokinetic and Phar-macodynamic Features of Nanoemulsion Following Oral, Intravenous, Topical and Nasal Route. Curr. Pharm. Des..

[92] Srivastava S., Mohammad S., Pant A.B., Mishra P.R., Pandey G., Gupta S., Farooqui S. (2018). Co-delivery of 5-Fluorouracil and Curcumin Nanohybrid Formulations for Improved Chemotherapy Against Oral Squamous Cell Carcinoma. J. Maxillofac. Oral Surg..

[93] Klang V., Matsko N.B., Valenta C., Hofer F. (2012). Electron microscopy of nanoemulsions: An essential tool for characterisation and stability assessment. Micron.

[94] Mohamadi L., Bazrafshan E., Noroozifar M., Ansari-Moghaddam A., Barahuie F., Balarak D. (2017). Adsorptive Removal of Benzene and Toluene from Aqueous Environments by Cupric Oxide Nanoparticles: Kinetics and Isotherm Studies. J. Chem..

[95] Ramana L.N., Sethuraman S., Ranga U., Krishnan U.M. (2010). Development of a liposomal nanodelivery system for nevirapine. J. Biomed. Sci..

